# A brief instrument measuring the water, sanitation and hygiene domain of menstrual health among women who inject drugs

**DOI:** 10.1371/journal.pone.0303378

**Published:** 2024-05-10

**Authors:** Alhelí Calderón-Villarreal, Lourdes Johanna Avelar Portillo, Daniela Abramovitz, Shira Goldenberg, Shawn Flanigan, Penelope J. E. Quintana, Alicia Harvey-Vera, Carlos F. Vera, Gudelia Rangel, Steffanie A. Strathdee, Georgia L. Kayser

**Affiliations:** 1 Herbert Wertheim School of Public Health and Human Longevity Science, University of California San Diego (UCSD), San Diego, California, United States of America; 2 School of Public Health, San Diego State University (SDSU), San Diego, California, United States of America; 3 Benioff Homelessness and Housing Initiative, School of Medicine, University of California, San Francisco, San Francisco, California, United States of America; 4 Division of Global Health, Herbert Wertheim School of Public Health and Human Longevity Science, UCSD, San Diego, California, United States of America; 5 Department of Medicine, Division of Infectious Diseases and Global Public Health, UCSD, San Diego, California, United States of America; 6 School of Public Affairs, SDSU, San, Diego, California, United States of America; 7 Universidad de Xochicalco, Tijuana, Baja California, Mexico; 8 El Colegio de la Frontera Norte, Tijuana, Baja California, Mexico; 9 Border Health Commission, Tijuana, Baja California, Mexico; University of New Mexico Health Sciences Center, UNITED STATES

## Abstract

**Background:**

Domains of adequate menstrual health (MH) include access to water, sanitation, and hygiene (WASH). People who menstruate with social disadvantages—such as homelessness or drug injection practices–often face barriers to WASH access. However, validated instruments to measure MH are limited among marginalized populations, and available instruments involve lengthy surveys. We developed and evaluated psychometric properties of a novel ‘*MH WASH Domain Scale-12*’ among people who menstruate and who inject drugs in the Tijuana–San Diego region and identified correlates of MH access using this scale.

**Methods:**

We constructed a MH-scale based on access to twelve WASH-related items: (1) menstrual products, (2) body hygiene (bathing per week), (3) water sources for bathing, (4) improved, (5) non-shared, (6) available, (7) private, (8) nearby, (9) and safe sanitation facilities, (10) availability of soap, (11) water source for handwashing, and (12) handwashing facilities with soap/water. Variables were dichotomized and summed, with scores ranging from 0–12 points and higher scores indicating better MH access. We assessed the scale’s reliability and construct and content validity using data from a binational cross-sectional study. The sample included people who inject drugs (PWID) who had ever menstruated in their lifetime and were 18+ during 2020–2021. MH-WASH items were described, and the scale was further used as an outcome variable to identify correlates.

**Results:**

Among 125 (124 cis-female and 1 trans-male) PWID that reported menstruating, our ‘*MH WASH Domain Scale-12’* was reliable (Cronbach’s alpha = 0.81, McDonald’s Omega total = 0.83) and valid. We identified two sub-domains: Factor-1 included items describing ‘WASH availability’ and Factor-2 contained items related to ‘WASH security’—encompassing physical and biological safety. Scale scores were significantly lower among participants experiencing unsheltered homelessness compared to participants experiencing sheltered homelessness or living in permanent housing.

**Conclusion:**

We constructed and validated a novel and reliable scale to measure MH-related WASH access that can be used to assess MH among marginalized populations in English- and Spanish-speaking contexts. Using this scale we identified disparities in MH-WASH access among PWID and who menstruate in the US-Mexico border region.

## Introduction

One important domain of menstrual health is body care–which requires access to water, sanitation, and hygiene (WASH). Menstrual health (MH) was defined in 2021 by the Global Menstrual Collective as “a state of complete physical, mental, and social well-being and not merely the absence of disease, in relation to the menstrual cycle” [1]. Achieving MH is based on a multi-domains construct that implies women, girls, and all people who menstruate have access to ‘information’, ‘health care’, ‘an environment free of stigma’, ‘participation in all spheres of life’, and ‘WASH facilities and services’ [1]. The WASH domain, also known as the ‘body care’ domain of MH, was defined for menstruating individuals as “care for their bodies during menstruation such that their preferences, hygiene, comfort, privacy, and safety are supported” [1]. This includes access to menstrual materials and private and safe facilities for changing, washing their body and hands (e.g., handwashing facilities with water and soap), and cleaning and/or disposing of used materials [1]. Of note, the WASH domain of MH is not referred to as ‘menstrual hygiene’, a term that has been argued to simplify and stigmatize menstruation as something unclean, but as a comprehensive approach of important elements for ‘caring for one’s body’ during menstruation—which is only one of the five main domains of MH [1,2].

PWID are a vulnerable group because of drug use, criminalization, many experience homelessness and many engage in sex work. Women, girls, trans-man and non-binary people who menstruate and who are engaged in substance use face barriers to access sexual and reproductive health and MH-WASH services [3–6]. Few studies have explored MH among PWID who menstruate and particularly few in the Tijuana-San Diego region [5,7]. Menstruation has been identified as an extra challenge among women who inject drugs and who experience homelessness [7]. In order to access MH, women who inject drugs and experience homelessness prioritize access to private and safe WASH facilities, menstrual products and washing their bodies more often [7]. Lack of private facilities can impact MH access among PWID experiencing homelessness [5]. Moreover, lack of MH-WASH services among PWID are often exacerbated by stigma, violence, and police victimization. Among women who inject drugs, unmet MH needs had been associated with self-isolation and feeling “disgusting” and “nasty”, and stigma is commonly reported during menstruation, limiting women’s access to facilities and resources [7]. Additionally, people with intersecting vulnerabilities such as those experiencing homelessness, people engaged in sex work, those living with socioeconomic/geographic disadvantages, and mobile populations often face additional challenges accessing those services which have been barely addressed with current MH scales [1,3,8,9].

To accurately track MH, the development and validation of novel measurements are needed to characterize and address the unmet needs of marginalized populations [10,11]. There are currently inconsistencies between definitions and operationalization of indicators used to measure MH needs, including the WASH domain of MH [10]. Access to menstrual management products is not enough to achieve menstrual health [12], and WASH services need be included when measuring MH access [11]. Few instruments that measure MH domains are currently available. One of the most important measurement schemata of MH developed to date was produced by the Joint Monitoring Programme (JMP) for Water Supply, Sanitation and Hygiene by the United Nations International Children’s Emergency Fund (UNICEF) and the World Health Organization (WHO). The JMP instrument considers educational and cultural components and a single aspect of WASH services (having water and soap available) [13]. However, the JMP also monitors WASH access in a separate component with lengthy questionnaires (100+ variables). Hennegan et al developed a comprehensive instrument to assess menstrual practice needs, including some WASH elements in 2020 [14]. This instrument included items to describe how comfortable or clean adults and adolescents feel managing their menstruation, and covered key WASH variables [14,15]. In 2023, Hennegan et al described an indicator shortlist to monitor girl’s MH and hygiene, including indicators focused on menstrual material access, stigma, health care and WASH services at school [16]. Although this instrument and instrument shortlist is useful for understanding women’s and girls’ needs in household and school contexts, it may not be suitable for marginalized populations if the duration of the survey application is long or focused on the household or school, particularly among unhoused or mobile populations for whom short surveys are more useful. To date, no instruments or brief scales have been developed for PWID, people experiencing homelessness, or mobile populations who menstruate or tested among these populations.

There is limited knowledge of MH among PWID and a need for novel measures that assess MH among marginalized population. The aim of this study was to develop and provide preliminary validation of a measure of the WASH domain of MH and to apply the scale to identify sociodemographic characteristics that are related to the MH-WASH access among people who menstruate and who inject drugs in the Tijuana–San Diego metropolitan area.

## Methods

### Dataset

Data used for this cross-sectional analysis was collected at a single study visit during 2020–2021 from participants enrolled in a prospective *La Frontera* cohort study of PWID who live in the binational metropolitan area of Tijuana, Baja California, Mexico and San Diego, California, United States (US). This study received ethics approval from the institutional review boards at UCSD in La Jolla, California (IRB #191390) and Xochicalco University in Tijuana, Mexico. Participants provided written informed consent. Monetary incentives (total of $45 USD per individual) were provided to participants who were enrolled in the study and completed the screening and supplemental visit (administered approximately one week after the baseline visit) visits. Recruitment period started on 10/28/2020 and ended on 10/25/2021.

The *La Frontera* cohort study used nonprobability sampling to identify individuals who reported injecting drugs within the last month (as evidenced through injection stigmata) and reported living in Tijuana or San Diego. Due to the main objective of the parent student, we enrolled participants residing in San Diego who reported having crossed the border to inject drugs in Tijuana within the last two years as well as those from either city who reported not having used illicit drugs on the other side of the border. Eligibility criteria for the parent study included individuals aged ≥ 18 or older who reported injecting drugs within the last month (verified by inspecting injection stigmata), living in Tijuana or San Diego between 2020 and 2021 [17]. Among the 612 participants enrolled in *La Frontera* cohort study participants who reported they had ever menstruated in their lifetime and completed the WASH component questionnaire were included in this analysis, and we excluded 24 participants who reported never having menstruated (primary amenorrhea).

Data were collected using street outreach and mobile vans in Tijuana and San Diego and surveys were administered by trained, bilingual interviewers who were residents of either city, as previously described in Strathdee et al., 2021 [17]. The survey components included in this analysis are sociodemographic characteristics and MH-WASH variables. Sociodemographic characteristics included city of residence, gender identity, age, hours spent outside, housing status, and sex work status.

### Descriptive statistics

We described general characteristics of the participants, including sociodemographic, MH-WASH variables, and menstrual status. We explored MH-WASH by sociodemographic characteristics and described MH-WASH access gaps between population subgroups based on chi squared (X^2^) significance testing. Menstrual status was classified into three categories: based on each participants’ last date of menstruation, and was classified as follows: menstruating, of reproductive age with amenorrhea (i.e., not menstruating), and menopause (age greater than 49-years-old without menstrual cycles). Menstruating status was based on self-reporting having menstruation in the past 12 months. Socioeconomic variables were utilized to describe the population, validate the scale, and identify correlates. These variables included city of residence, gender identity, age, hours spent outside (on the streets), housing status, and sex work status. Age was a continuous variable. City of residence included San Diego and Tijuana. Gender identity included women and one trans man as we only studied people who had ever menstruated. Sex work in the last six months was a dichotomous variable, based on whether or not the study participant reported ‘prostitution or sex work’ as one of their sources of income during the specified time period. Housing status was classified into three categories: permanent housing, sheltered homelessness and unsheltered homelessness, as defined by the US Department of Housing and Urban Development [18,19]. In this study, housing status was defined as the main place where a participant slept in the last six months. Permanent housing included participants who reported sleeping in a house or apartment owned or rented by participants’ parents, themselves, their spouse/sexual partner, family, or friends. Sheltered homelessness status included a participant reported having slept in a migrant worker camp, asylum shelter, shelter/welfare residence, workplace, short-term rented room (hotel, motel, or other rooming house), deportee shelter/camp, correctional institution (jail, prison, detention center), drug treatment center, medical care facility (i.e., hospital, hospice, or nursing home), or rented garage. Unsheltered homelessness status was categorized as participants who slept in a car, bus, truck, or other vehicle, abandoned building, on the streets, beach, parks, canal, woods, and shooting gallery in the last six months.

### The ‘MH WASH Domain Scale-12’

The constructed *‘MH WASH Domain Scale-12’* contained MH access related items from a list of 22 WASH variables, including use and access to WASH services and MH materials. To develop the scale, we first explored pairwise correlations to select the items with positive or neutral correlations using Kendall’s correlation. The items included menstrual products used during the last period; sanitation (toilet) facilities that were improved, non-shared, available, private, nearby, and safe; having more than four showers/baths in the last week; improved water sources for bathing; and available soap (for any use), water sources for handwashing, and handwashing facilities with soap and water (Table 1). MH products access included reusable and disposable sanitary pads, tampons, and menstrual cups. Water sources and toilet facilities items referred to the main source/facility used in the past six months and were categorized as improved based on the JMP definitions [13]. Improved water sources for showering/bathing included private and public tap water, protected well and spring, rainwater, delivered water, and packaged water. Improved toilet facilities include flush or pour flush toilet, pit latrine with slab, and composting toilet. A non-shared toilet refers to a toilet that was not shared with other households or with the general public. Sanitation privacy was defined as the frequency participants used toilets that were private (i.e., with a functioning door and lock) and classified as ‘always/usually available’ vs. ‘sometimes/rarely/never’. Nearby toilet was defined as a toilet facility accessible within 10 minutes or less walking distance based on the average distance walked in the sampled population. A safe sanitation facility was defined by the participants as not having experienced any physical, sexual, or verbal assault, robbery, arrest, or other form of harassment and violence while using the toilet. Availability of services/facilities was defined as ‘always or usually’ available. All variables were dichotomized because not all the individual variables had the same unit of measurement in the survey, where ‘1’ corresponds to an affirmative response (representing access), and 0 to a negative response (representing no or limited access).

**Table 1:** Items included in the ’*Menstrual Health WASH Domain Scale-12*’.

Variable	Dichotomous Responses
Menstrual products used in the last period	1 = Reusable sanitary pads/disposable sanitary pads/ tampons/menstrual cup; 0 = Underwear/toilet paper/cloth/nothing
Having 4+ showers in the last week	1 = >4 showers; 0 = <5 showers
Improved water sources for bathing	1 = Improved; 0 = Unimproved/surface water/no water sources
Improved toilet facility	1 = Improved; 0 = Limited/unimproved/open defecation
Non-shared toilet	1 = Not shared; 0 = Shared with more than one household/general public
Toilet privacy (door and lock)	1 = Always/usually available toilet with a door and lock; 0 = Sometimes/rarely/never available toilet with a door and lock
Toilet nearby	1 = Spent <10 minute walking; 0 = 10 minutes walking
Safe toilet (free of violence experiences)	1 = Did not experience harassment or violence using the toilet; 0 = Did experience physical, sexual or verbal assault, robbery, arrest, or other type of harassment/violence
Toilet available (open and not locked)	1 = Always/usually; 0 = Sometimes/rarely/never
Soap available for any use	1 = Always/usually; 0 = Sometimes/rarely/never
Water sources for handwashing available	1 = Always/usually; 0 = Sometimes/rarely/never
Handwashing facility with soap and water available	1 = Always/usually; 0 = Sometimes/rarely/never

To validate the *‘MH WASH Domain Scale-12’*, we required well-defined domains (i.e., smaller conceptual pieces feeding the construct) [20]. To define these domains, we assessed the degree to which the selected items were good measures of the construct. To assess the degree to which these items measured what they were supposed to measure, we conducted a parallel analysis and an exploratory factor analysis. Based on the variance (h2) and correlations per each item we defined the best number of domains to organize the construct. The items with the most variability were included in the MH-WASH scale.

### Validity and reliability of the ‘MH WASH Domain Scale-12’

To validate the scale, we performed validity and reliability assessments to test if the scale was accurately measuring the defined domain and how well it performed [20]. First, to assess the validity of the scale, we tested if the scale was indeed measuring the WASH domain of MH. We conducted two validity tests: content and construct. Content validity assessed if the items adequately measured the domain of interests [21]. Content validity (i.e., theoretical analysis) was tested in four stages: 1) defined theoretically the items and domains based on a literature review, 2) conducted a parallel analysis and exploratory factor analysis, 3) explored the relationship between each item in the construct and in the novel two-factor grouping, and 4) theoretically redefined the novel factor distribution (domains) [20,21].

To evaluate the theoretical relationship of the scale with other constructs, we conducted construct validity testing [20]. Construct validity testing assess the correlation of the scale and its domains/sub-scales with other variables [21–23]. Construct validity was tested using Kendall’s correlations. The sub-scales demonstrated covariation with other variables in accordance with the type of relationship we expected from the theoretical framework [20]. We selected constructs/variables not used in the scale to explore positive, negative and neutral associations [21]. Three variables were used to construct validity in our scale: ‘toilet functionality’, ‘hours spent outside’ (on the streets), and ‘age’. It is assumed that our scale items were positively correlated to other WASH items (not used in these scales), such as ‘toilet functionality’, because based on the literature we know that ‘sanitation access’ and ‘water source access’ variables must be related with a functional toilet facility [24,25]. Also, ‘hours spent outside’ (on the street) was expected to decrease WASH access, which was negatively related to ‘housing status’; that means, women without formal housing spend more time outside and have less access to WASH facilities [26]. Finally, ‘age’ in years and WASH access were not associated in the literature.

Additionally, to assess the reliability of the scale, we evaluated the internal consistency (i.e., the degree to which the set of items in the scale co-vary, relative to their sum score). We divided all variations of the scale into the unique and shared variation and computed the proportion of total variation that was shared. This assessment used two metrics: Cronbach’s Alpha (α) and McDonald’s Omega total (ω). We estimated α, the most common (traditional) and generally agreed upon internal consistency test [21]. However, α has four assumptions that limit their use: each scale item must contribute equally to the total score, the items of the scale should be normally distributed, there should be no correlations between the error of the items, and the scale should be unidimensional [20]. To address α limitations, we used the coefficient ω, which was an estimate of the general factor saturation of a test, allowing for the use of smaller subdomains to organize items after accounting for the primary domain [20]. The cutoff point was ≥ 0.80 for both reliability tests [21]. We described the scale and sub-scales distribution and measures of central tendency: mean, median, and mode, with the corresponding measures of dispersion, standard deviation (SD) and interquartile range (IQR).

### Sociodemographic variables and correlates of the ‘MH WASH Domain Scale-12’

We evaluated city of residence, sex work, and housing status as potential correlates of the *‘MH WASH Domain Scale-12’* score. Since the score was highly skewed to the left, to facilitate an appropriate inference method, we first converted the score into a 0–1 interval variable by dividing the scale score by 12, with the new variable essentially representing the proportion of score items to which a participant had access to. Then, this new variable was used as the outcome variable in a Beta regression model to evaluate its association with independent variables. We conducted univariate and multivariable regression analyses between the independent (sociodemographic variables) and dependent variables (scale score). We calculated unadjusted and adjusted risk ratios (RR, aRR) along with the corresponding 95% confidence intervals (CIs) using R version 4.0.2.

## Results

### Sample characteristics

Of the 125 participants (124 cis-females and 1 trans-male) who reported they had ever menstruated in their lifetime; the average age was 42.7 (SD = 10.4). Sixty-one percent (n = 76) of participants lived in San Diego, and the remaining 39% (n = 49) lived in Tijuana. Among those included in this study, 42% reported experiencing unsheltered homelessness at least once in the last six months. In the study, 32% reported currently experiencing unsheltered homelessness, 30% reported experiencing sheltered homelessness, and 38% reported living in permanent housing (Table 2). Twenty-seven percent of participants reported sex work in the last six months. Of all participants who reported ever menstruating in their lifetime, 57% were menstruating (i.e., have had at least one menstruation in the last 12 months), 17% were of reproductive age without menstruation (i.e., secondary amenorrhea), and 26% were in menopause. Secondary amenorrhea was not experienced by any of the participants who reported sex work, but it was experienced by 23% of participants who did not report sex work (p = 0.005). The prevalence of secondary amenorrhea was 24% in San Diego residents and 6% among those living in Tijuana (p = 0.02).

**Table 2:** Sociodemographic and WASH characteristics of 125 PWID and who have menstruated participants in the Tijuana-San Diego metropolitan area in 2020–2021.

Variable	n	Value
Age mean (SD)	125	42.7 (10.4)
Gender (%)		
Women	124	99.2
Trans men	1	0.8
City of residence (%)		
Tijuana	49	39.2
San Diego	76	60.8
Housing status (%)		
Main housing in the last 6 months		
Permanent housing	48	38.4
Sheltered homelessness	37	29.6
Unsheltered homelessness	40	32
Unsheltered homelessness at least once in the last 6 months	52	41.6
Number of hours spent outside per day mean (SD)	125	13.3 (7.7)
Sex work in the las 6 months (%)		
No	91	72.8
Yes	34	27.2
Menstrual status (%)		
Menstruating	71	56.8
Reproductive age with amenorrhea 12+ months	21	16.8
Menopause	33	26.4
Availability WASH variables		
Handwashing facility with soap/water available	87	69.6
Soap available for any use	94	75.2
Water source for handwashing available	106	84.8
Menstrual products used in the last period	106	84.8
Toilet available	112	89.6
Security WASH variables		
Non-shared toilet	52	41.6
Toilet privacy	89	71.2
Improved toilet facility	102	81.6
Safe toilet (free of violence)	105	84.0
Toilet nearby (<10 min walking)	108	86.4
Having >4 showers in the last week	78	62.4
Improved water source for bathing	123	98.4
Total	125	100

### MH-WASH characteristics

In this study, we also examine variables that capture the WASH domain of MH. In our sampled population, 15% of participants reported lacking access to menstrual products during their last period, 38% reported showering less than five times in the last week, and 2% reported water lacking or using unimproved/surface water as their main water source for bathing. Fifteen percent of participants reported no access to water sources to practice daily handwashing, 30% had no handwashing facilities with soap and water available all the time, and 25% had no soap always/usually available for any use. In terms of sanitation access, 10% of participants did not have access to toilet facilities, 29% relied on sanitation facilities that were not private (with a working lock and door), 18% that were not improved, and 14% reported walking over 10 minutes to reach a toilet facility. Furthermore, 16% of participants experienced violence or harassment using a toilet. Among those individuals who reported experiencing violence while using a toilet (N = 20), the most common types of violence were verbal (75%) and physical assault (40%) (Table 2).

Most of the MH-WASH access indicators varied by housing status. A higher proportion of participants experiencing unsheltered homelessness shared their main toilet facility (83%) in comparison to those individuals experiencing sheltered homelessness (49%) and those who lived in permanent housing (45%, X^2^ p<0.001). Two-thirds (65%) of people experiencing unsheltered homelessness used a public toilet as their main toilet facility (sharing it with the general public), whereas only 16% of participants experiencing sheltered homelessness and 13% of those living in permanent housing did so (p<0.001). Only half (53%) of participants experiencing unsheltered homelessness had access to toilet facilities that had privacy, whereas individuals experiencing sheltered homelessness and those living in permanent housing had 76% and 85% access, respectively (p<0.001). Similarly, access to improved toilet facilities was lower (65%) among individuals experiencing unsheltered homelessness than among sheltered participants (86%) and those living in permanent housing (94%, p<0.001). Only 43% of participants experiencing unsheltered homelessness had access to more than four showers per week, while more than 70% of the participants that reported experiencing sheltered homelessness and 74% of permanently housed participants had the corresponding access (p = 0.007). Availability of handwashing facilities with soap and water was lower among participants experiencing homelessness (p = 0.023), particularly among those experiencing sheltered homelessness (unsheltered = 65%, sheltered = 57%), in comparison with those living in permanent housing (85%). We also found a social gradient by housing status for availability of soap, as those participants experiencing unsheltered homelessness (65%) and sheltered homelessness (70%) had lower access to soap (for any use) than those living in permanent housing (89%) (p = 0.037). We found that harassment or violence experienced while using toilet facilities had a social gradient based on housing status, with only 9% of participants living in permanent housing reporting ever experiencing harassment or violence while using the toilet, compared to 14% of those experiencing sheltered homelessness and 28% of those experiencing unsheltered homelessness (p = 0.045). Among sheltered and unsheltered homeless individuals who had experienced violence, verbal assault was the most common type (sheltered = 80%, unsheltered = 64%), followed by physical assault (sheltered = 40%, unsheltered = 36%).

We also found that MH-WASH needs differed significantly by sociodemographic variables. PWID living in Tijuana had lower access to menstrual products compared to those living in San Diego (71% vs 93%, respectively p = 0.002) and lower access to available handwashing facilities with soap and water (Tijuana: 55% vs San Diego: 79%, p = 0.009). Participants who reported sex work in the last six months had lower access to available water for handwashing (71% vs 90%, p = 0.015) and to handwashing facilities with soap and water (50% vs 77%, p = 0.007), as compared to those who did not report sex work.

### The ‘MH WASH Domain Scale-12’

**Fig 1:**
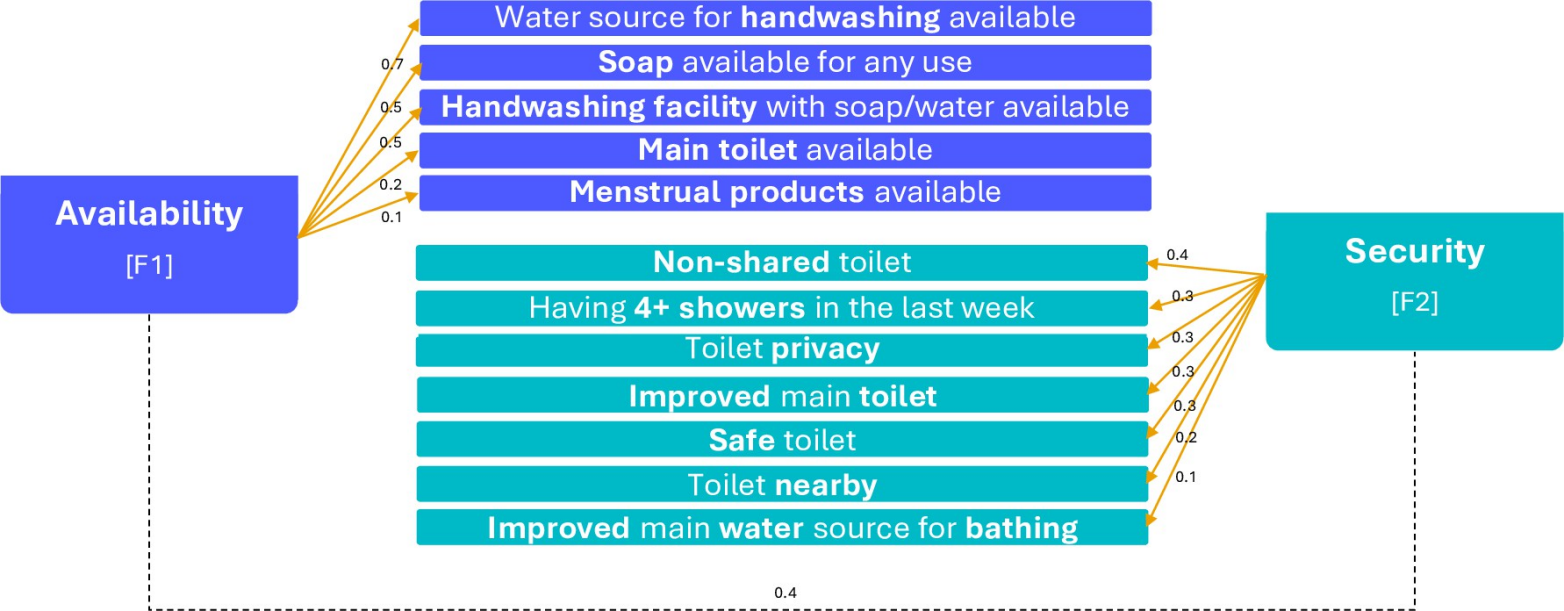
Factor analysis of the *‘Menstrual Health WASH Domain Scale-12’* items.

For the construction of the ‘*MH WASH Domain Scale-12’*, twelve items with mainly positive or neutral correlations were included. We identified that using two-factors (referred to as F1 and F2) best organized the construct (S1 Table) based on parallel and factor analyses. The identified factors corresponded to two sub-domains. Factor-1 (F1) included five items (water for handwashing available, soap available for any use, handwashing facility with water and soap available, main toilet available, and menstrual products) broadly related to the availability of WASH sources, and consequently we defined it as the *‘WASH Availability’* sub-scale (Fig 1). Factor-2 (F2) contained seven items related to *‘WASH Security’*—encompassing physical and biological safety—including improved water sources for bathing, and access to improved, nearby (<10 min walking), non-shared, private, and safe toilet facilities. These sub-scale items contained two areas: a) the security of the water used (for bathing) as defined by appropriateness for human consumption or use without health risk and b) physical security accessing sanitation facilities. In sum, this process resulted in two sub-scales: ‘*WASH availability*’ and ‘*WASH security*’.

Correlations between sub-scales to ‘toilet functionality’ (F1 = 0.35, F2 = 0.24) were positive, which could be interpreted as both sub-scales being similarly correlated to ‘toilet functionality’. Both sub-scales were negatively correlated to ‘hours spent outside’ (F1 = -0.10, F2 = -0.35). However, the *WASH Security* sub-scale had a stronger correlation with the ‘hours spent outside’, which could be related to the lack of access to appropriate sanitation and bathing facilities outside a household. No correlations were found with respect to ‘age’ (F1 = 0.03, F2 = 0.05). These findings were in agreement with the assumptions taken from the theoretical framework.

**Fig 2:**
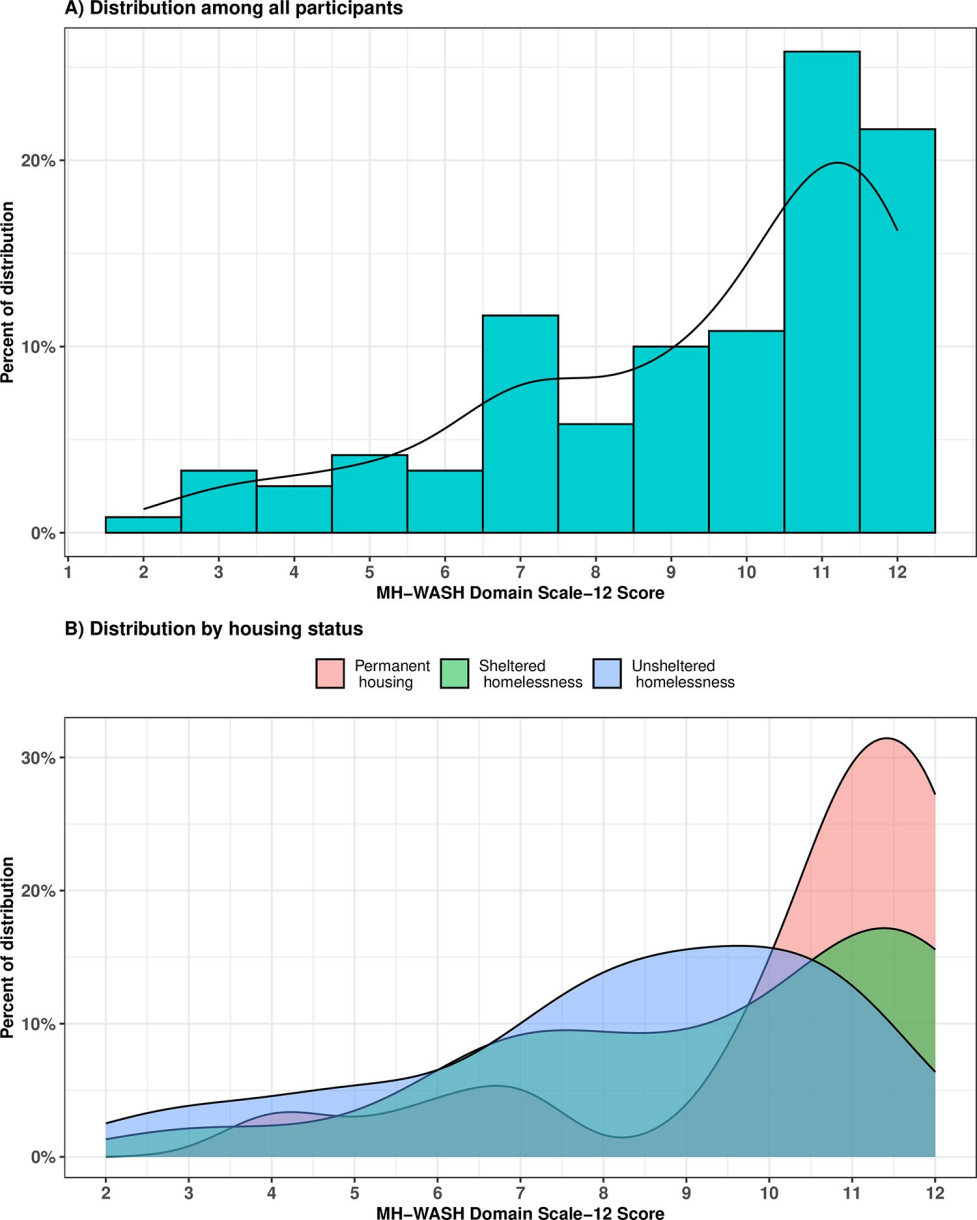
*‘Menstrual Health WASH Domain Scale-12’* score distribution among 125 PWID and who have menstruated in Tijuana-San Diego metropolitan area.

Among 125 participants that reported ever menstruating, our ‘*MH WASH Domain Scale-12’* was found reliable (α: 0.80, ω total: 0.83) and valid. Omega total’s reliability was more appropriate for our scale characteristics, yet both traditional (α) and alternative (ω) reliability methods showed consistent findings. The ‘*MH WASH Domain Scale-12’* score had a minimum value of 0 and a maximum value of 12, with a higher score indicating higher access to MH-WASH. The distribution of the score was skewed to the left, mostly because 59% of participants had a scale score of 10+ points (Fig 2). The score mean was 9.4 (SD = 2.6), a median of 10.0 (IQR = 7.7–11.0), and a mode of 11.0. The ‘WASH Availability’ sub-scale score had a minimum value of 0 and maximum of 5, score mean of 4.1 (SD = 1.4), median of 5 (IQR = 3–5), and mode of 5. The ‘WASH Security’ sub-scale score had a minimum value of 0 and maximum of 7, score mean of 5.3 (SD = 1.6), median of 6 (IQR = 4–7), and mode of 7.

### Correlates of the ‘MH WASH Domain Scale-12’

The MH-WASH score was significantly associated with housing status (Fig 2). Participants living in permanent housing (aRR: 3.18, CI95%: 2.00, 5.04) as well as those experiencing sheltered homelessness (aRR: 2.76, 95%CI: 1.65, 4.63) had significantly higher access to MH-WASH as compared to participants experiencing unsheltered homelessness (*MH-WASH Domain-12* score: 10.2 [SD = 2.3], 9.4 [SD = 2.6], 8.2 [SD = 2.6] respectively) (Table 3). We found association by city of residence only after adjusting for covariates, San Diego residents had significantly higher access to MH-WASH (aRR: 1.61, CI95%: 1.03, 2.52) than those residing in Tijuana (Tijuana 8.9 [SD = 2.9], San Diego 9.6 [SD = 2.4]). No associations were found by sex work status. People who menstruate and who inject drugs had comparable MH-WASH access by reported sex work in the past six months (sex work 9.0 [SD = 2.7], no sex work 9.5 [SD = 2.5]).

**Table 3:** Correlates of the ’*Menstrual Health WASH Domain Scale-12*’ among 125 PWID and who have menstruated in Tijuana-San Diego metropolitan area.

Variable	Level	WASH-MH scale score (SD)	RR	95% CI	aRR	95% CI
City of residence	Tijuana	8.9 (2.9)				
	San Diego	9.6 (2.4)	1.40	0.94, 2.10	1.61	1.03, 2.52*
Sex work status	Yes	9.0 (2.7)				
	No	9.5 (2.5)	1.22	0.79, 1.87	1.01	0.62, 1.65
Housing status	Unsheltered homelessness	8.2 (2.6)				
	Sheltered homelessness	9.4 (2.6)	2.36	1.46, 3.80*	2.76	1.65, 4.63*
	Permanent	10.2 (2.3)	3.06	1.93, 4.85*	3.18	2.00, 5.04*

* Significant value.

## Discussion

In this study, we report on reliability, validity, and correlates of a brief, novel scale to assess the WASH domain of MH among women who menstruate and who inject drugs, which was found to be both reliable and valid. We identified two sub-scales–*WASH availability* and *WASH security*–whose performance was assessed. Our scale complements the available ‘Menstrual Practices Questionnaire’ (MPQ) and ‘Menstrual Practices Needs Scale’ (MPNS-36) validated by Hennegan et al. (2020) in Uganda. The MPNS-36 and MPQ are longer and more detailed instruments including variables about perceptions of comfort, satisfaction, adequacy, worries and concerns during the last menstrual period, and about activities undertaken to collect, contain, and removed menstrual blood from the body [14,15]. These instruments are useful for women and girls, especially in households and school environments. Particularly for girls, the multi-dimensional ‘Menstrual Hygiene Management Scale’ (MHHM) validated in India is useful to explore behaviors related to preparation of clean absorbent, hygiene, and disposal in households and school environments [27]. The available MH instruments focused on a variety of MH domains mainly in Asian and African contexts [14,15,27]. Yet, limited literature has examined or measured MH and/or MH-WASH domain in the Americas region, and none in binational contexts.

An important strength of this study is that this scale was based on the WHO/UNICEF JMP definitions for WASH. The JMP is the largest, nationally representative global effort for WASH monitoring, making our scale and its variables comparable and easy to adapt to populations from different contexts. The JMP also has an important MH instrument and others available to measure WASH. The JMP instrument for MH considers mainly educational and cultural components, and WASH indicators are explored in different contexts by extensive WASH questionnaires separately [13]. Our scale provides a briefer alternative to explored WASH services linked with MH, with fewer items, which does not take long to administer and is easy to apply to other marginalized populations. Additionally, our scale includes variables not considered by the JMP, such as measuring WASH beyond the household unit of measurement, the time taken to access sanitation facilities and frequency of access to showers/baths to maintain body hygiene.

WASH insecurity disproportionately affects women [28,29]. Women’s WASH needs are different than men due to biological differences such as menstruation and pregnancy [8,30]. Women’s access to WASH services, together with other MH related actions such as elimination of menstrual product taxes, menstrual education, and campaigns against stigma, are all important elements to promote gender equity [12,28,31]. Assessments of MH-WASH access represent measurements of gendered WASH access that pertain to the Sustainable Development Goal targets 6.1 and 6.2 for WASH access among women and girls [28]. Caruso et al and the JMP recently proposed 15 WASH indicators for enhanced monitoring of gender in WASH, some also relate to MH [28]. The proposed 2023 sanitation and hygiene indicators included a clean, private and safe sanitation facility, not feeling unsafe at sanitation facilities due to fear of being harmed or assaulted, and access to sufficient menstrual materials and to a clean, private, and safe space during their last menstrual period, which are similar to four of the items included in our scale (sanitation facility that is private, non-shared and free of violence or harassment, and access to menstrual materials) [28]. However, several of the aforementioned indicators for gender in WASH are based on the household level, limiting their use among people experiencing homelessness or housing insecurity. Further, this draft MH-WASH JMP indicators do not include time to sanitation, which is an important measure of access for sanitation that impact MH. Furthermore, our scale overlaps with the WASH gender equality measure (WASH-GEM) developed by Carrard et al, is also at the individual level, and our scale is compatible with its ‘resources’ domain [31]. Yet, measures and indicators of gender in WASH, such as the WASH-GEM, explore a larger set of domains related to gender equity, and are beyond the MH objective of our study, but should be considered in future studies of MH among vulnerable populations.

Our scale provides one of the few assessments of an important domain of MH access. The scale was validated among PWID and who reported ever menstruating. PWID represent a marginalized population underexplored by the WASH and MH fields, which often experience insecurity accessing basic services–including WASH [32]. Marginalized populations often share common sources of structural oppression limiting their access to WASH services. We developed a scale that is not linked with substance use or housing characteristics but instead focuses on WASH insecurities experienced among PWID and how menstruate along the US-Mexico border region. The scale validated in this study can subsequently be validated and used among other populations to test the MH-WASH domain, which may be particularly useful among marginalized and mobile populations (e.g., unhoused, migrants, and refugees), PWID in other locations, trans men who inject drugs, and communities experiencing WASH insecurity. Furthermore, our scale is shorter than other available instruments, taking less time to measure and easy to be included in longer surveys, complementing the current set of available instruments to measure MH.

The exploration of demographic variables expands our understanding of intersecting vulnerabilities affecting menstrual health access particularly among women who menstruate and who inject drugs. These findings complement Keiser et al.’s study on menstrual health needs among 62 women of reproductive age on treatment for substance use disorder in Virginia, that reported menstrual poverty in 81% of the participants [3]. Scale scores were correlated with housing status. Access to a household or a shelter was significantly associated with higher scores on our ‘*MH WASH Domain Scale-12’*, highlighting the MH-WASH insecurity unsheltered women who menstruate and who inject drugs experience on a daily basis. Our results complement the studies conducted in Los Angeles and New York City (NYC) on menstruation and homelessness. Avelar Portillo et al.’s study among 79 women and 2 transgender men experiencing homelessness in Los Angeles, reported insecurity accessing MH-WASH services: 27% reported insecurity accessing >4 showers per week, while in our study 38% reported this challenge [6]. Gruer et al.’s (2021) study among individuals experiencing homelessness in NYC, reported that people experiencing sheltered and unsheltered homelessness often experience insecurity accessing menstrual products, yet the most vulnerable were people experiencing unsheltered homelessness who are less likely to gain access to these products [33]. Maroko et al. conducted a mixed-methods study also in NYC among people experiencing homelessness, and found that access to clean, private, and available sanitation facilities are critical issues and unmet needs among this population [34]. Sommer et al. qualitative study also among NYC people experiencing homelessness, reported lack of clean and safe spaces to manage menstruation, and inadequate bathing and laundering access [35]. Yet, our study is one of the first to describe MH among PWID.

There are some limitations to this study. Study sampling was nonprobability sampling, given the difficulty of reaching the target population. Therefore, caution must be used when interpreting results or generalizing. We used the same population to validate the scale and to identify correlates, which could incorporate bias. Most of the variables in this scale were based on well-defined concepts. However, some variables had less defined cutoffs points, such as number of baths per week and time spent walking to sanitation facilities. These two variables have not been well explored in past literature, but we argue are important for MH access and for mobile populations who do not have permanent housing and limited access to these services. Permanent housing in this study included all participants who were not classified as individuals experiencing homelessness, but should not be interpreted as someone with stable long-term housing. Rather, we focused on the place or structure where participants reported sleeping, which can provide some access to WASH and protect them from the elements. However, this does not imply participants may have permanent access to that infrastructure. Participants who were classified as living in permanent housing could also be experiencing housing instability in ways not explored in this analysis. The participant dataset included only one trans man, which limited the validation and results of this study to the experiences of cis women who inject drugs. We included two cities that are part of a binational metropolitan area; however, due to the sample size, we were unable to assess if the scale offers similar reliability in both locations. Also, because we used latent variables that included self-reported information, our findings could have been influenced by social desirability or motivation biases [22]. Moreover, this scale aimed to measure one of the five domains of MH and cannot measure MH comprehensively.

Recent studies are developing scales to include measures of the other four components of MH focused on menstrual education, health care access, respectful environment and freedom to participate in all spheres of life during menstruation [1,16]. MH was defined in 2021[1] and the JMP Gender in WASH short list of indicators that will be measured across countries was developed by Hennegen et al in 2023 [16]. Our data used in this MH-WASH scale was collected in 2020 and 2021 while these other scales were developed. Our MH-WASH scale’s strengths are in its brevity, important for studies that do not focus on MH across all five domains, and it is tested in a vulnerable population, PWID who are largely unhoused, a population rarely studied in MH research. Our scale could be tested in other mobile or vulnerable populations, like refugees, internally displaced people, asylum seekers and returnees [36].

MH is an essential part of health for women and people who menstruate. However, we found that access to WASH services needed to secure MH is not universal among PWID living in the Tijuana-San Diego region. In our study, we found that less than 80% of participants had access to >4 showers/baths per week, had available handwashing facilities (with soap and water) and soap for any use, and access to improved, non-shared, and private toilet facilities. We identified gaps in access to WASH services important for achieving MH, which requires us to shift our focus and think about the implications of housing status among PWID. For instance, availability of handwashing facilities with soap and water was identified as an important need among individuals experiencing homelessness, participants who reported sex work, and those living in Tijuana. Additionally, improvements are needed to bring access to bathing and safe, private, and non-shared sanitation facilities for participants experiencing both sheltered and unsheltered homelessness. Availability of improved water sources for handwashing should be increased among participants living in Tijuana and those who reported sex work.

The prevalence of secondary amenorrhea (i.e., absence of menstrual cycles for 12+ months) among women of reproductive age is up to 3% (ranging between 1.0% to 5.0%) in different contexts [37–39], and in our study among PWID, we found 17% of participants reported secondary amenorrhea. Our study supports previous research reporting that substance use is a risk factor for amenorrhea [40,41]. Harlow et al. (2003) estimated injection drug use nearly quadrupled the risk of amenorrhea among 1075 women aged 20–44 years in the US (3%), while among our study participants prevalence of amenorrhea was almost six times that of the general population of women of reproductive age [40]. Further, in Kaiser et al.’s study among women of reproductive age enrolled on substance use disorder treatment, 42% reported secondary amenorrhea, which was more than double that in our study [3]. Menstrual cycles can be affected by body weight changes, chronic illness, eating and exercise habits, stress, and medication use [42,43]. Despite the association between substance use and amenorrhea, it is important to ensure medical care among women, non-binary, and transgender men of reproductive age without menstrual cycles to identify and treat physiological causes such as premature menopause, polycystic ovarian syndrome, or hyperprolactinemia [42]. The ‘health care’ domain of MH defined as the access to “diagnosis, treatment, and care for menstrual cycle-related discomforts and disorders, including access to appropriate health services and resources, pain relief, and strategies for self-care” is highly important among PWID who menstruate and should be explored in future research [1].

## Conclusions

We constructed and validated, in a US-Mexico cohort population, a novel and reliable scale to measure the WASH domain of MH that can be used to assess MH among marginalized populations including women who inject drugs and who are experiencing homelessness in English- and Spanish-speaking contexts. According to the validity assessments employed in this analysis, the ‘*MH WASH Domain Scale-12’* contained two domains: *WASH availability* and *WASH security*. The performance of these sub-scales was assessed theoretically and empirically using a suite of available statistical tools. Overall performance was deemed to be adequate, and further research should be conducted to validate and compare this scale and its derived sub-scales among other marginalized populations, such as non-binary and trans men who menstruate who also experience WASH insecurity and other mobile populations in emergency setting or are experiencing housing insecurity. This is a short and easy-to-use scale which may be useful for rapid assessment and for inclusion in longer surveys. Access to a household or a shelter was significantly associated with higher scores on our ‘*MH WASH Domain Scale-12’* which highlights the importance of housing security to achieve MH.

We identified specific WASH needs by housing status, city of residence, and reported sex work among PWID that require further attention. Moreover, women who inject drugs of reproductive age had high prevalence of amenorrhea, which requires medical care to rule out other diagnoses. Interventions to improve access to MH-WASH services are needed among women who menstruate and who inject drugs, particularly for bathing, available handwashing facilities and soap, and improved, non-shared, and private sanitation facilities.

## Supporting information

S1 Table Variance and correlation per item by two factors among the ‘*Menstrual Health WASH Domain Scale-12’* items. (DOCX)

S1 File (CSV)

S1 Database (CSV)
