# Supplementary material for: A brief instrument measuring the water, sanitation and hygiene domain of menstrual health among women who inject drugs
Source: PLoS One. 2024 May 10;19(5):e0303378. doi: 10.1371/journal.pone.0303378 (PMC11086918; doi:10.1371/journal.pone.0303378)
Supplement: S1 Table — (DOCX) [file pone.0303378.s001.docx]

**Supplementary Table 1.** Variance and correlation per item by two factors among the ‘*Menstrual Health WASH Domain Scale-12’* items

| **Variable** | **Factor-1** | **Factor-2** | **h2** |
| --- | --- | --- | --- |
| Water source for handwashing available | **0.67** | -0.07 | 0.88 |
| Soap available for any use | **0.47** | 0.16 | 0.72 |
| Handwashing facility with soap/water available | **0.46** | 0.08 | 0.56 |
| Toilet available | **0.24** | 0.10 | 0.20 |
| Menstrual products | **0.09** | 0.07 | 0.04 |
| Non-shared toilet | 0.02 | **0.39** | 0.35 |
| Having >4 showers in the last week | -0.04 | **0.34** | 0.23 |
| Toilet privacy | 0.28 | **0.32** | 0.62 |
| Improved toilet facility | 0.06 | **0.32** | 0.27 |
| Safe toilet | -0.07 | **0.31** | 0.17 |
| Toilet nearby | 0.01 | **0.23** | 0.12 |
| Improved water source for bathing | 0.11 | **0.13** | 0.10 |

h2 – variance
